# Unmasking pandemic patterns: decoding the COVID-19’s impact on mortality in Italy with Generalized Gamma overdispersion model

**DOI:** 10.1186/s12889-025-25036-6

**Published:** 2025-11-21

**Authors:** Danila Azzolina, Rosanna Comoretto, Daniela Ferrante, Corrado Magnani, Dario Gregori

**Affiliations:** 1 Department of Translational Medical Science, Biostatistics and Clinical Trial Methodology Unit, Clinical Research Center DEMeTra, University of Naples Federico II, Naples, Italy; 2 Department of Pediatrics and Public Health, University of Turin, Turin, Italy; 3 Department of Translational Medicine, University of Eastern Piedmont, Novara, Italy; 4 Department of Cardiac Thoracic, Vascular Sciences, Unit of Biostatistics, Epidemiology and Public Health, University of Padua, Via Loredan 18, Padua, 35131 Italy

**Keywords:** Excess mortality, COVID-19, Italy, Generalized gamma model

## Abstract

**Background:**

Italy has been significantly impacted by the COVID-19 pandemic, particularly in its early stages, resulting in healthcare strain, societal restrictions, and disruption. Understanding the long-term effects, notably excess mortality beyond the initial peaks, remains important. Prior studies have focused on the early phase, leaving out subsequent and updated mortality trends.

**Method:**

This study analyzes Italian mortality rates from 2015 to 2023, employing Generalized Additive Models for Location, Scale, and Shape (GAMLSS), the Generalized Gamma Overdispersion model. Data analysis considered factors such as gender, age groups (under 65 and 65 or older), and geographical differences (Northern versus Central-Southern Italy) as key characteristics of the mortality trend.

**Results:**

The study identified several phases of the pandemic, characterized by a significant early 2020 mortality peak and subsequent smaller peaks. Mortality rates were higher in Northern Italy, with males and the elderly being the most affected. Overall, mortality rates increased during the pandemic, particularly among these groups, and then returned to normal levels in 2023. An increase in the overdispersion parameter, estimated via the GAMLSS model, is evident in the post-pandemic phase and persists until 2023.

**Conclusion:**

The findings highlight the complex nature of COVID-19’s impact on mortality in Italy. They reveal the temporal phases, regional disparities, and demographic vulnerabilities that contribute to the overall mortality picture. The overdispersion component indicates more significant variability and unpredictability of mortality patterns until 2023. This highlights the intricate interplay of factors, including healthcare capacity, viral mutations, and the effectiveness of public health responses. This study emphasizes the need for targeted interventions and protective measures in the most affected groups.

**Supplementary Information:**

The online version contains supplementary material available at 10.1186/s12889-025-25036-6.

## Background

The COVID-19 pandemic, caused by SARS-CoV-2, was a major global health crisis. Italy, as the European epicenter, faced overwhelmed healthcare systems, strict social restrictions, and significant disruptions to daily life [1, 2]. Excess mortality is an essential indicator for understanding the impact of the COVID-19 pandemic on the population, as it exceeds the official death toll attributed directly to the virus [3]. In Italy, several studies have investigated excess mortality during the COVID-19 period, shedding light on the dynamics and extent of the pandemic’s contribution to the population’s mortality rate, especially in the first year of the pandemic. Michelozzi and colleagues analyzed mortality data from various regions in Italy during the early months of the pandemic and compared them to mortality rates in the same periods in previous years [4]. Furthermore, Magnani et al. [5] evaluated the extent of total excess deaths during the COVID-19 pandemic in Italy during the first trimester. Data from 4,433 municipalities providing mortality reports up to April 15, 2020, were included, covering a total of 34.5 million residents from all Italian regions. Data were analyzed by region, sex, and age and compared to the expected data from the 2015–2019 pre-pandemic period. Findings from both studies revealed a significant spike in excess mortality, coinciding with the peak of the pandemic, which exceeded the number of reported COVID-19 deaths. This suggests that the virus may have indirectly contributed to the high number of deaths, possibly due to pressure on healthcare systems, resulting in limited access to medical care for other diseases and disruptions in routine health services [4, 5].

Several researchers have conducted multinational comparisons of excess mortality during the COVID-19 pandemic. Kontis et al. (2020) conducted a multinational study encompassing 21 industrialized countries, including Italy, to assess the overall effect of the first wave of the COVID-19 pandemic on all-cause mortality [6]. Their analysis demonstrated that Italy experienced a substantial increase in all-cause mortality during the pandemic period, exceeding the expected mortality levels based on historical trends. The study highlighted the significant impact of the virus on excess deaths and suggested that the pandemic’s toll extended beyond the direct effects of COVID-19 infections, also *“because of increased mortality from other diseases due to reductions in acute and chronic care”* [6].

Furthermore, the literature demonstrates that understanding the demographic and health-related factors associated with COVID-19 mortality is important for implementing targeted interventions and resources to protect those at higher risk. A study conducted by Onder, Rezza, and Brusaferro (2020) investigated the case-fatality rate and characteristics of patients dying from COVID-19 in Italy. This study highlighted the severity of the virus in vulnerable populations, including the elderly and those with pre-existing health conditions [7]. Moreover, research on this topic has shown that the excess mortality in 2020 is mainly due to male [8] and elderly subjects [5].

These studies collectively underline the importance of studying excess mortality during the COVID-19 pandemic in Italy. They revealed the multidimensional impact of the pandemic on mortality patterns, encompassing not only COVID-19-related deaths but also indirect consequences on healthcare and vulnerable populations. Such results are valid for public health decision-makers in tailoring effective strategies, allocating resources, and devising mitigation measures for future pandemics or health emergencies [9].

Excess mortality during Italy’s pandemic has been less examined in recent years. This extended analysis helps to understand the long-term effects that persist beyond the pandemic. This study, which analyzes mortality data up to December 2023 post-pandemic phase, aims to understand the impact of COVID-19 and enhance the response to future crises. It assesses the resilience of the healthcare system and identifies improvement needs, providing insights relevant to global public health for countries facing similar challenges, such as the UK and Spain [10].

## Materials and methods

### Data

The Italian National Statistics public repository (ISTAT) served as a source for mortality data categorized by municipality, age, and sex from 2015 to December 31, 2023, a period that coincided with an early post-pandemic phase, which could have been influenced by prior pandemic-related factors. Italian mortality data were collected for a total of 7,901 municipalities, encompassing the overall country’s resident population [11].

The resident population was obtained from the Demographics-Istat repository [12]. COVID-19-related deaths were sourced from the Italian Civil Protection Department [13] and processed using the data warehouse of Padua University [14]. These COVID-19-specific mortality statistics were available exclusively in the form of daily mortality counts, organized by region, and lacked any breakdown by age or sex.

In Italy, COVID-19 fatality is defined based on a positive result from the RT-PCR testing procedure without factoring in any pre-existing comorbidities. These statistics were used to describe the daily mortality figures attributed to COVID-19 [7] and to depict the daily mortality figures associated with COVID-19 in the present analyses.

The age groups were differentiated into two categories: 0–64 and 65+, as suggested by existing research on the lethality of COVID-19 by age, as well as prior investigations undertaken by the authors of this paper [5, 8]. Moreover, the Italian regions were arranged into predefined groupings using an a priori’ approach, making comparisons between the Northern Italy Regions (Piemonte, Valle d’Aosta, Lombardia, Trentino-Alto Adige, Veneto, Friuli Venezia Giulia, Liguria, and Emilia-Romagna) and the remaining regions of Italy. This classification is consistent with the distinct mortality patterns observed in Italy, particularly during the initial wave of the pandemic [5].

### Indicators

Mortality indicators were calculated for the entire country and categorized by region and sex. Specifically, these metrics include the following:

1. Overall mortality is defined as the daily mortality rate per 100 000 residents in the periods 2015–2019, 2020, 2021, 2022, and 2023 (until December 31); the average per year has also been reported.
2. The death rate attributable to COVID-19 is defined as the COVID-19 daily mortality rate per 100 000 residents in the periods 2015–2019, 2020, 2021, 2022, and 2023 (until December 31); the average per year has also been reported. The data has been reported only for the overall country and region, as it is not reported in the official statistics per gender and age classes.
3. The *P*-Score measures excess mortality as the percentage difference between the reported and expected number of deaths. The expected number of deaths was calculated as the mean of the deaths in the 2015–2019 pre-COVID-19 period as follows:

$$\begin{aligned}&\mathrm P-\mathrm{Score}=\;\frac{\mathrm{Reported}\;\mathrm{Deaths}\;-\;\mathrm{Expected}\;\mathrm{Deaths}}{\mathrm{Exapected}\;\mathrm{Deaths}}\;\ast\;100\end{aligned}$$

For example, if a region had a *P*-Score of 100% in a given period, the death count for that period was double the expected death count [15]. The daily *P*-Score and average per year have been reported.

4) The Differential of Mortality over the COVID-19 deaths was defined as the ratio of the excess to the reported COVID-19 deaths:

$$\begin{aligned}&\mathrm{Ratio}\;\mathrm{of}\;\mathrm{Excess}=\;\frac{\mathrm{Reported}\;\mathrm{Deaths}\;-\;\mathrm{Expected}\;\mathrm{Deaths}}{\mathrm{COVID-19}\;\mathrm{Deaths}}\end{aligned}$$

The data has been reported only in aggregated form per country and region, as it is not reported in the official statistics per gender and age classes.

As previously explained, official COVID-19 data are available only for the total population; therefore, indicators 2) and 4) are not reported for age- and sex-stratified mortality results. The daily distributions of these indicators were reported graphically. The indicators for Italy, regions, and sex by period were reported with 95% bootstrap confidence intervals from 1000 runs, as indicated in the literature [15].

### Data modelling

The mortality rate data were modeled by considering a Generalized Additive Model for Location, Scale, and Shape (GAMLSS) parameterization [16]. A Generalized Gamma random variable was considered to parameterize the distribution of the response variable, which is defined by skewed, positive values. The Generalized Gamma distribution, within the GAMLSS framework, was selected for its flexibility in modeling positively skewed, non-negative data, such as daily mortality rates. Unlike more restrictive distributions (e.g., the Poisson or standard Gamma), the Generalized Gamma allows for independent modeling of location, scale, and shape parameters, offering greater adaptability in capturing complex mortality patterns, particularly in the presence of overdispersion and asymmetry. This choice was further motivated by the need to estimate both central trends and variability across time, demographic strata, and geographic regions. The Generalized Gamma’s ability to encompass other distributions (e.g., it includes the Weibull and standard Gamma as exceptional cases) made it a suitable candidate for detecting subtle shifts in mortality dynamics across different phases of the pandemic [17].

The overall daily data were modeled to estimate the impact of timing, geographical area (Northern Italy vs. Central-Southern Italy), sex, and age on excess mortality. However, to account for an extra source of variability (overdispersion) in the mortality data, the period was included as a covariate on the scale component of the model. The log link is used to model both location and scale.

The exponentiated coefficients with 95% confidence intervals were reported. The exponentiated coefficient, obtained from the GAMLSS Generalized Gamma model, on the location parameter is interpreted as the Mortality Rate Ratio (MRR).

Exponentiated scale parameters have also been reported; this quantity is related to the dispersion or variability of the mortality data. If the exponentiated coefficient is greater than 1, it indicates an increase in the dispersion of the mortality data during the considered period compared to the pre-COVID-19 period (2015–2019), thereby widening the spread of the data. If the exponentiated coefficient is less than one, it indicates a decrease in dispersion, leading to a narrower distribution of mortality data. The Supplementary Material section provides additional technical details regarding the model.

Analyses were performed using R, version 4.3.2 [18]. The GAMLSS model was implemented using the gamlss package (version 5.4–20) [19].

## Results

### Italian data

**Fig. 1:**
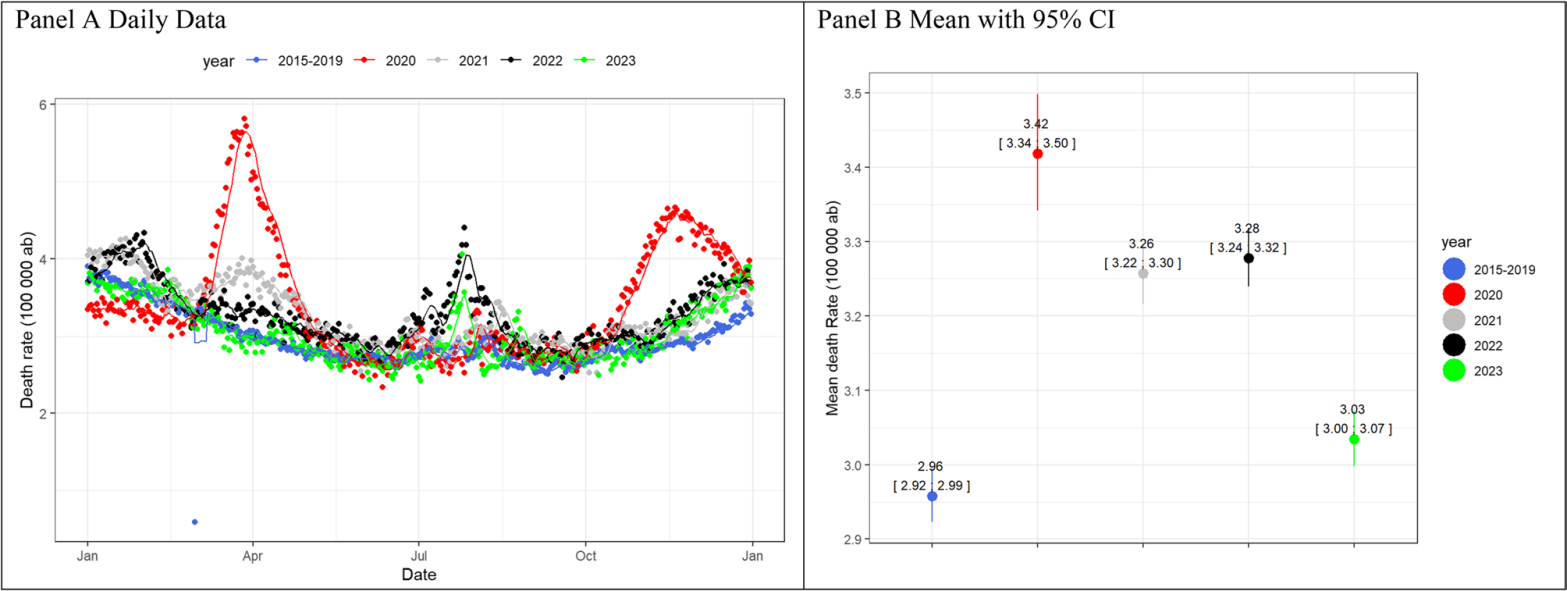
Mortality rate over 100 000 inhabitants (**ab**) for daily data (panel **A**) and mean with 95% bootstrap CI for the period January-December

In the Italian data, the overall mortality rate in 2020 reached its highest peak between February and May (Fig. 1, Panel A), followed by another surge from October 2020 to January 2021. The following years saw smaller peaks, with 2021 featuring two peaks in January and February, and 2022 having minor peaks in February and March, as well as in August and late in the year. The 2023 followed the 2015–2019 baseline average in mortality (Fig. 1, Panel B).

**Fig. 2:**
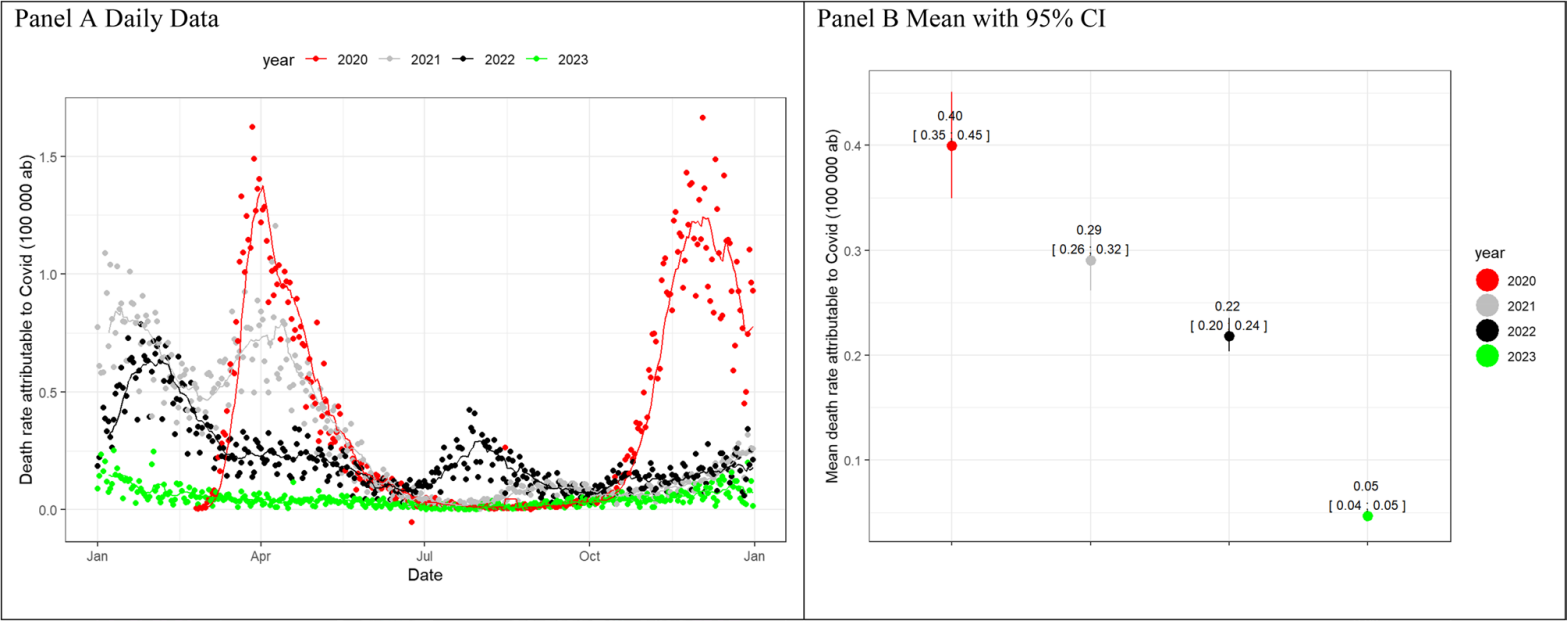
COVID-19 death rate over 100 000 inhabitants(**ab**) for daily data (panel **A**) and mean with 95% Bootstrap CI for the period January-December

**Fig. 3:**
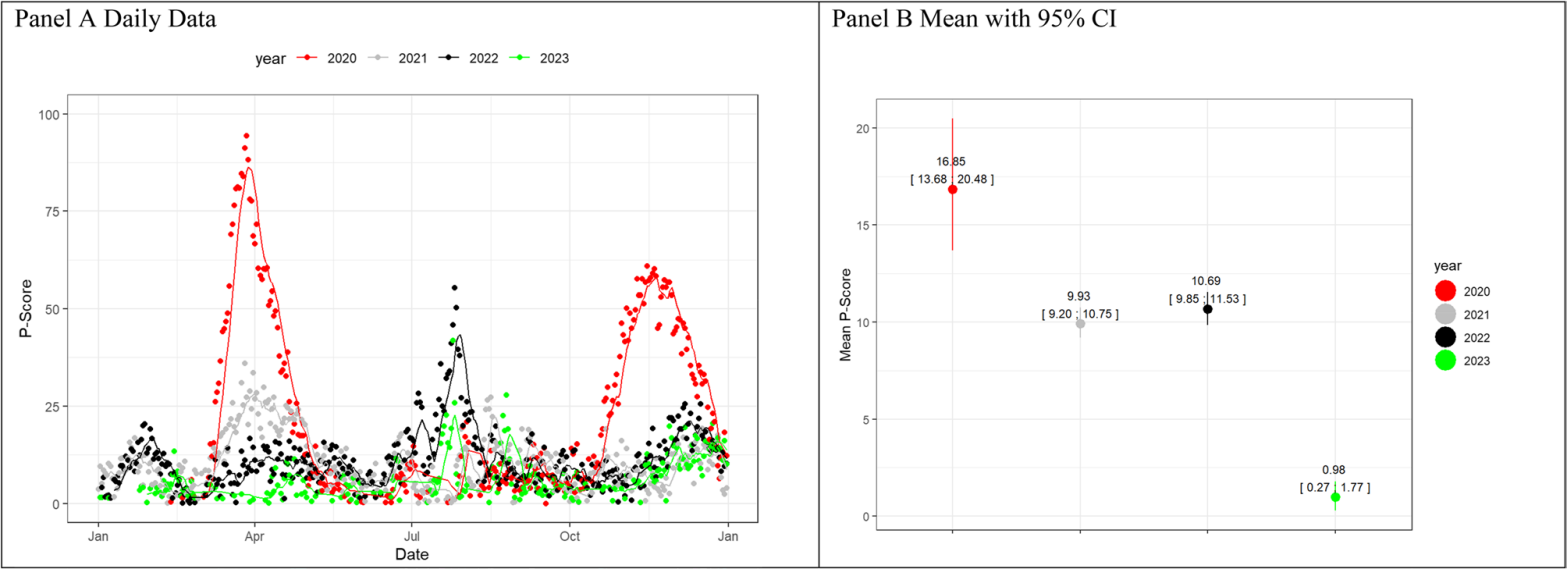
P-Score for daily data (panel **A**) and mean with 95% bootstrap CI for the period January-December

The COVID-19 mortality rate mirrored these trends, decreasing in 2023 (Fig. 2, Panel A). A gradual decline was observed from 2020 to 2023 (Fig. 2, Panel B). P-Score curves, reflecting excess mortality, showed initial peaks exceeding 50% compared to expected deaths from 2015 to 2019, but later years (2021–2023) had smaller peaks, staying below 50% (Fig. 3, Panel A). A remission of excess mortality has been shown in early post-pandemic in 2023 (Fig. 3, Panel B).

### Comparison across regions

**Fig. 4:**
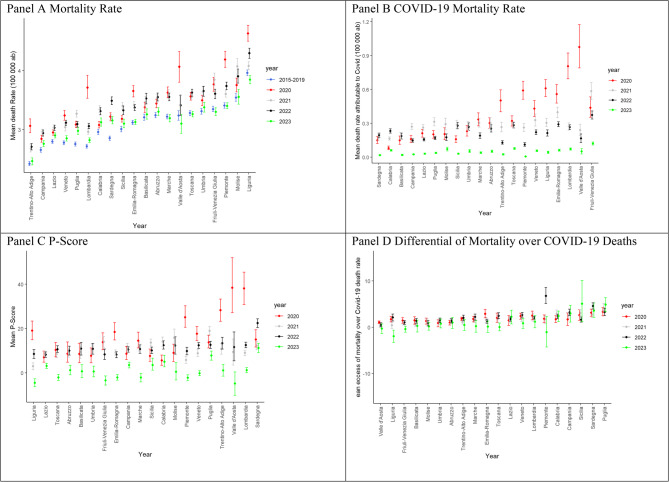
Regional means and 95% bootstrap CI according to years in the period January-December

The map in Fig. S1 shows the Italian regions divided into Northern and Southern regions. Until October 2020, higher mortality rates were observed in Northern Italy, particularly in Lombardia, Emilia-Romagna, Valle d’Aosta, Piemonte, and Liguria, with slightly lower rates in Trentino-Alto Adige, Friuli-Venezia Giulia, and Veneto (Fig. 4, Panel A).

In 2020, northern regions, such as Lombardia, Piemonte, Liguria, and Valle d’Aosta, experienced a higher increase in COVID-19 mortality. By 2021, increased rates were also seen in Central and Southern Italy, including Puglia and Molise (Fig. 4, Panel B).

The P-Score indicated excess mortality until October 2020, particularly in northern regions such as Lombardia, Trentino-Alto Adige, Emilia-Romagna, Piemonte, and Valle d’Aosta. Central and southern areas, such as Puglia and Molise, experienced higher excess mortality in 2021 (Fig. 4, Panel C). The mortality differential due to COVID-19 deaths was most noticeable in 2020 across all regions, both from January to December and for the entire year (Fig. 4, Panel).

### Sex-age stratification

The analysis of overall mortality by sex revealed consistent mortality peaks for both males and females, reflecting general population trends. However, males experienced more pronounced mortality peaks during the initial year of the pandemic, particularly in April and May, and at the end of the year (Fig. S2). This trend was also observed for excess mortality using the P-Score metric (Fig. S3). Between 2020 and 2022, the majority of overall mortality occurred in individuals aged 65 and older, with males exhibiting more pronounced mortality peaks than females (Fig. S4), a pattern also evident in the P-Score analysis (Fig. S5). For those under 65 years, the excess mortality did not exceed 50% of the 2015–2019 baseline mortality. In contrast, mortality in the elderly population has more than doubled in the early stages of the pandemic.

### Data modeling

Applying the GAMLSS model to the pandemic mortality dataset for the first eight months revealed a nationwide increase in mortality from 2021 to 2023. In 2020, the early pandemic period, this increase was significant only in Northern Italy, as compared to the 2015–2019 average. In Northern Italy, a decrease in mortality was observed in 2021 and 2023. The interaction effect revealed that in 2020, mortality rates were higher in the northern regions compared to the southern areas. The model also estimated higher Mortality Rate Ratios (MRR) for males and those aged 65 and older, with the overdispersion parameter indicating increased volatility in mortality during the pandemic (Table 1).

**Table 1:** GAMLSS generalized gamma model estimation. The model has been estimated for the January-December period. The 95% confidence intervals (CI) have been reported for MRR. The parameters have been reported for the location and scale (overdispersion) component of the model

	Predictors	MRR	CI	*P*-value
Location Parameters	Year [2020]	1.0525	1.0365–1.0688	< 0.001
	Year [2021]	1.099	1.0816–1.1166	< 0.001
	Year [2022]	1.0606	1.0437–1.0778	< 0.001
	Year [2023]	1.0514	1.0215–1.0821	0.001
	Area [North]	0.9344	0.9234–0.9454	< 0.001
	Sex [m]	1.3745	1.3637–1.3854	< 0.001
	Age Group [> = 65]	29.1308	28.8981–29.3654	< 0.001
	Year [2020] × Area [North]	1.1127	1.0887–1.1371	< 0.001
	Year [2021] × Area [North]	0.9626	0.9411–0.9846	0.001
	Year [2022] × Area [North]	0.9953	0.9729–1.0182	0.685
	Year [2023] × Area [North]	0.9498	0.9119–0.9893	0.013
Dispersion Parameters	Year [2020]	1.5553	1.4997–1.6129	< 0.001
	Year [2021]	1.6386	1.5804–1.6988	< 0.001
	Year [2022]	1.6565	1.5974–1.7178	< 0.001
	Year [2023]	2.1364	2.0387–2.2387	< 0.001

## Discussion

This study thoroughly examined mortality trends in Italy during the COVID-19 pandemic, investigating how factors such as time, location, sex, and age affected mortality rates. It traces these trends from the onset of the pandemic to early post-pandemic in 2023, highlighting their dynamic evolution in Italy, one of the countries severely affected by the pandemic.

While 2023 no longer represents the peak of the pandemic, it remains influenced by its long-term effects, including lingering health consequences, healthcare disruptions, and socioeconomic factors that impact mortality trends [20]. For this reason, extending our analysis to 2023 provides an essential context for evaluating how mortality patterns have evolved, comparing pandemic years (2020–2022) with pre-pandemic baselines (2015–2019) and the post-pandemic stabilization phase. This assessment helps determine whether mortality returns to baseline or reflects lasting changes, offering indications for public health resilience and informing future pandemic preparedness.

These findings are consistent with international research, which shows a similar excess COVID-19 mortality ratio in Italy, as observed in global data from October 2020 to February 2022 [3].

The first two years of the pandemic revealed international similarities and differences in excess mortality, highlighting the common challenges faced by global healthcare systems. The primary among these was the direct impact of COVID-19, including the strain on healthcare facilities and resources. This often results in delayed or inadequate care for both COVID-19 and non-COVID-19 patients, leading to higher mortality rates [5]. Indirect effects, such as delayed care for chronic conditions, mental health issues from extended lockdowns, and socioeconomic disparities, have also significantly impacted mortality rates. In countries with more significant socioeconomic gaps, the pandemic has hit underprivileged communities harder, resulting in higher mortality rates [3]. Public health strategies and their effectiveness in controlling the spread of viruses play important roles. Variations in lockdown measures, testing capacities, and vaccination campaigns across countries and at regional levels have led to divergent outcomes in managing their impacts [21].

This study presents an updated temporal analysis of overall mortality rates up to 2023, providing a compelling narrative of the pandemic’s progression in Italy. The year 2020, serving as the pandemic’s inaugural year, is characterized by a stark peak in mortality between February and May [5], followed by a secondary surge from October to January. However, subsequent years revealed a series of smaller mortality peaks. The year 2021 exhibited two minor peaks in January and February, whereas 2022 showed a peak in August and another peak at the year’s conclusion [22]. By the first eight months of 2023, the post-pandemic period, the mortality curve in Italy began to align with the 2015–2019 baseline, indicating a stabilization of the pandemic’s impact on mortality. This highlights the distinct temporal phases of the pandemic and underscores the importance of vigilant monitoring of excess mortality. Understanding the multi-wave nature of the pandemic is crucial for anticipating surges, adjusting resource allocation, and implementing interventions to protect healthcare systems and vulnerable populations [23]. The study’s P-Score analysis reveals peaks in excess mortality during the pandemic, followed by a decline from 2021 to 2023, indicating a reduction in the pandemic’s impact on mortality. This trend reflects the effectiveness of improved public health policies, the evolution of COVID-19 treatments, and improved healthcare coordination [21]. Moreover, the widespread and extensive rollout of vaccines has likely contributed to reducing the severity and spread of the virus, complementing public health policies, evolving treatment protocols, and enhancing healthcare coordination [24].

The interplay between COVID-19 mortality rates and general mortality trends is a noteworthy finding [3]. The congruence of the two sets of rates, evidenced in this study, underscores the interdependence of the pandemic’s consequences on the broader mortality landscape. Moreover, the observed reduction in the average COVID-19 mortality rate between 2021 and 2023, mainly from January to May, suggests that the health system and public health measures may have had more opportunities to improve the organization and management of healthcare facilities in addressing the impact [25]. This trend is consistent with previous research indicating the positive influence of interventions such as vaccination campaigns, improved clinical protocols, and public health messaging in reducing COVID-19 mortality rates over time [26].

A series of challenges and adaptive responses have characterized Italy’s experience during the pandemic. The initial waves in 2020 led to a steep learning curve, prompting the country to institute strict lockdown measures and prioritize testing and contact-tracing efforts [1]. Moreover, public health campaigns emphasizing preventive measures, such as social distancing, mask-wearing, and hygiene practices, have contributed to reducing the spread of the virus and, consequently, the burden on healthcare systems [27]. Subsequent years saw a refinement of these strategies, as Italy capitalized on lessons learned from the earlier stages of the pandemic. The 2021 vaccination rollout played a crucial role in reducing the severity of COVID-19, resulting in fewer hospitalizations and deaths, particularly among vulnerable groups, and in enhancing the capacity of healthcare systems to manage the pandemic [28].

Northern Italy, particularly regions such as Lombardia and Emilia-Romagna, experienced high mortality rates, with peaks in early and late 2020. Southern Italy saw lower and later peaks in 2020. The early emergence of COVID-19 in wealthier northern regions of Italy may be partially explained by higher levels of international mobility and participation in activities such as alpine tourism and skiing, which may have facilitated the virus’s early spread before widespread containment measures were implemented [29].

The Marche region experienced an early peak, likely influenced by nearby areas that were heavily affected. Another exception is the observed delayed and lower mortality excess in the Veneto region in 2020, compared to other northern regions [30]. One possible explanation is the region’s proactive approach to implementing stringent public health measures early in the pandemic period. Veneto was among the first Italian regions to adopt aggressive containment strategies, including extensive testing, contact tracing, and isolation protocols, which may have contributed to curbing the initial spread of the virus and consequently mitigating the immediate mortality impact [31]. Furthermore, the presence of a robust healthcare infrastructure and a well-coordinated response system in Veneto may have played a role in mitigating the pandemic’s impact [32].

The study revealed that males experienced higher mortality peaks than females in the initial year of the pandemic, particularly in April and May, and at the end of the year, indicating increased male vulnerability. This sex-based difference in mortality is further supported by the P-Score analysis and the existing literature [8]. Similarly, subjects under 65 years of age experienced less pronounced excess mortality, reinforcing the pandemic’s disproportional impact on the elderly population [33]. The less pronounced excess mortality among subjects under 65 years of age could be attributed to differences in vulnerability and prevalence of comorbidities. The elderly population often has a higher incidence of chronic conditions, such as diabetes, obesity, and other comorbidities, which exacerbate the severity of COVID-19 and increase the risk of fatal outcomes. Younger individuals, who typically have fewer health issues, are generally less susceptible to severe complications from the virus [34].

The use of the GAMLSS model reinforced these findings by providing a quantitative analysis of pandemic-induced mortality shifts. A notable public health observation is the increase in the overdispersion parameter, particularly in 2020, which suggests greater variability and unpredictability in mortality patterns. This highlights the intricate interplay of factors such as healthcare capacity, viral mutations, and the effectiveness of public health responses. This highlights the necessity for flexible strategies that can adapt to the evolving nature of the pandemic’s impact, ensuring a comprehensive and effective public health approach to protecting population health [1, 35, 36].

The findings of this study contribute to the understanding of the mortality dynamics during the COVID-19 pandemic in Italy. A multifaceted analysis of temporal trends, regional disparities, and demographic vulnerabilities offers insights for policymakers, researchers, and public health practitioners. Multidimensional exploration, along with the application of advanced statistical models, contributes to a comprehensive 

### Study limitations and future research developments

The lower COVID-19-attributed mortality in Southern Italy may, in part, be due to under-detection of cases. This aligns with previous studies highlighting significant regional variations in testing capacity and reporting during the pandemic, particularly in the context of socioeconomic disparities, such as those observed in Italy [9, 10, 15]. Studies such as Msemburi et al. [15] and Wang et al. [3] have shown that the under-detection of COVID-19 cases is often more pronounced in regions with limited healthcare resources or during periods of high healthcare system burden. Furthermore, Dorrucci et al. [9] noted that testing policies and healthcare infrastructure played an important role in revealing the observed geographic disparities in Italy during the pandemic. The literature also highlights the likelihood that Northern Italy’s higher COVID-19 mortality rate is influenced by increased detection and reporting, due to its better-equipped healthcare systems and more widespread testing availability [30, 31].

Socioeconomic differences are well-documented as significant contributors to variations in COVID-19 outcomes. For example, regions with higher incomes and better healthcare access generally have lower mortality rates, while underserved areas often experience delayed treatment and higher fatality rates [37, 38]. Similarly, air pollution, particularly in industrialized regions such as Northern Italy, is known to exacerbate the severity of respiratory diseases, including COVID-19 [39]. However, analysis reported in this manuscript relies on official macro-regional national statistical data, which, while sufficient for high-level patterns, does not allow for detailed investigations into confounding factors such as socioeconomic disparities [37, 38], cause-specific mortality [9], or environmental influences such as air pollution [39]. The inclusion of these factors was not feasible because of the lack of regionally disaggregated and consistently available daily data for the entire study period. Future research should aim to integrate these important determinants as more granular and comprehensive datasets become accessible.

Another limitation stems from reliance on all-cause mortality data. The literature reported that the pandemic indirectly influenced mortality from other causes, such as cardiovascular diseases, respiratory illnesses, and cancer, owing to delayed treatments and healthcare access restrictions [5, 8, 37]. In contrast, this study’s primary contribution lies in its macro-regional perspective, which offers a high-level view of mortality trends across time. More detailed data are required to understand the effects of the pandemic on cause-specific mortality fully.

Moreover, while this study provides an analysis of excess mortality trends, it does not capture the broader consequences of the COVID-19 pandemic, such as the reduction in quality of life, long-term disability, and mental health outcomes. These aspects, although beyond the scope of this work, are critical for health policy and should be the focus of future studies aiming to comprehensively assess the pandemic’s burden and inform resilient public health strategies.

Despite these limitations, our study represents an essential step toward understanding and reconstructing the evolving mortality impacts of the COVID-19 pandemic over time, until the early post-pandemic phase, and provides a framework to guide future research. Additional research efforts are necessary to more accurately characterize COVID-19 mortality patterns at the patient level, including cause-specific mortality, socioeconomic indicators, and environmental factors.

## Conclusion

This study unveils pandemic-induced mortality patterns in Italy, revealing the temporal phases, regional variations, and demographic vulnerabilities. The GAMLSS model underscores the increased mortality during the pandemic, while the sex-age analysis highlighted disparities. Although 2023 may be characterized as a post-pandemic phase, the persistent elevation in mortality variability and marginal excess mortality indicates ongoing challenges for the healthcare system. These findings underscore the need for sustained mitigation strategies to manage the after-effects of the pandemic, including the treatment of long COVID, the restoration of disrupted care pathways, and the reinforcement of public health infrastructure. The study’s findings should inform the development of tailored interventions to inform effective public health strategies in case of pandemic outbreaks.

## Supplementary Information

Supplementary Material 1.

## Data Availability

The datasets generated and/or analysed during the current study are available from the corresponding author on reasonable request.
