# Supplementary material for: Unmasking pandemic patterns: decoding the COVID-19’s impact on mortality in Italy with Generalized Gamma overdispersion model
Source: BMC Public Health. 2025 Nov 21;25:4401. doi: 10.1186/s12889-025-25036-6 (PMC12754894; doi:10.1186/s12889-025-25036-6)
Supplement: Supplementary file 1 — Supplementary Material 1. [file 12889_2025_25036_MOESM1_ESM.docx]

# Supplementary Material

## Generalized Gamma

The Generalized Gamma distribution, sometimes called the Weibull-Gamma distribution, is often used to model positive continuous data that follows a skewed pattern. The Partial Density Function (PDF) is given by:

$$f(y\mid\mu,\sigma)=\frac{y^{\left( 1/\sigma^{2} \right)-1}\cdot\exp\left( -\frac{y}{\left( \sigma^{2} \right)\cdot\mu} \right)}{\left( \sigma^{2}\cdot\mu\right)^{1/\sigma^{2}}\cdot\Gamma\left( \frac{1}{\sigma^{2}} \right)}$$

Where:

- $y$ is the random response variable.

*μ* is a positive parameter that affects the location of the distribution. The parameter corresponds to the distribution mean

- *σ* is a positive scale of the distribution. This quantity is the square root of the usual dispersion parameter for a GLM Gamma model. Hence *σ μ* is the standard deviation of the distribution
- $\Gamma()$ is the gamma function.

## Additional findings

| 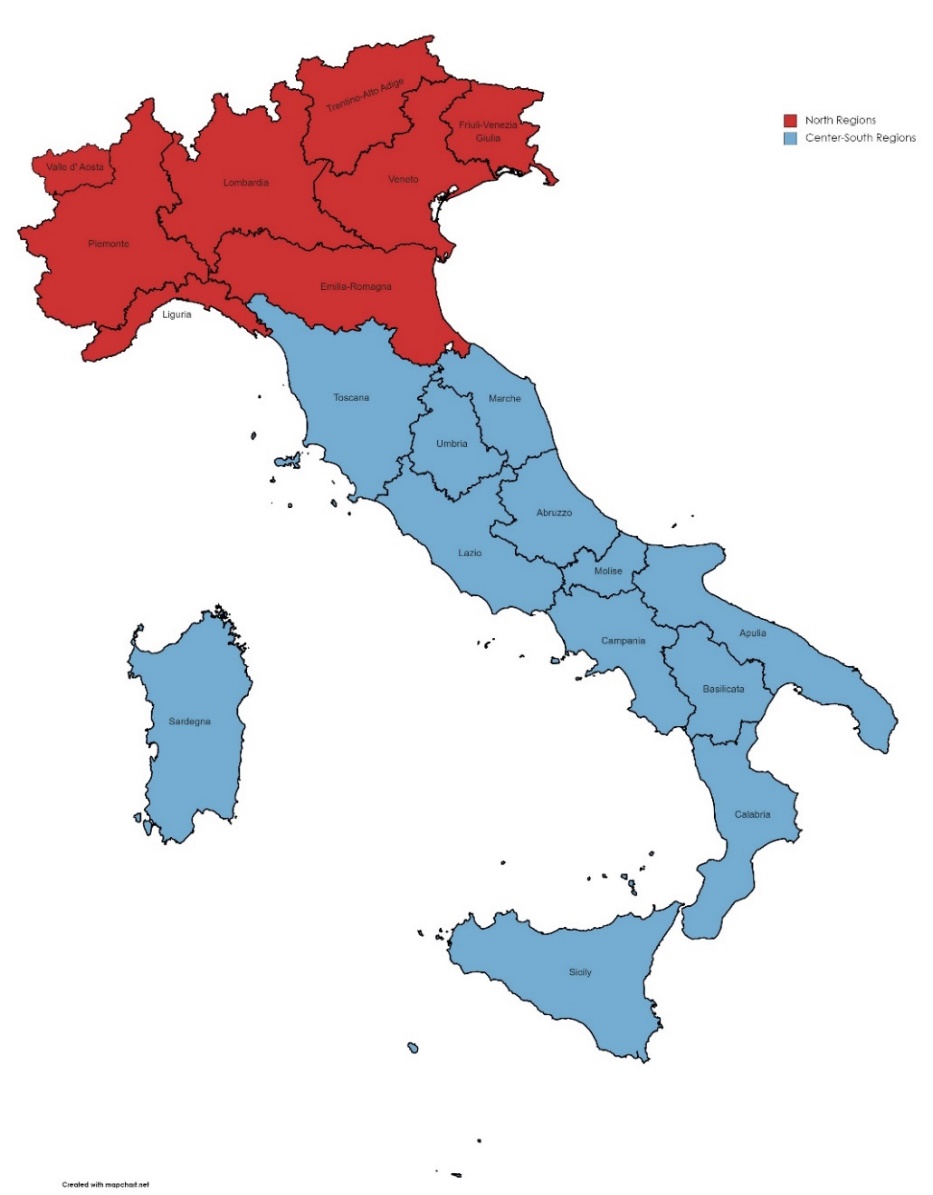 | **Northern Regions:** Liguria, Piemonte, Valle D'Aosta, Lombardia, Emilia-Romagna, Trentino Alto-Adige, Veneto, Friuli-Venezia Giulia  **Central-Southern Regions:** Lazio, Marche, Toscana, Umbria, Abruzzo, Basilicata, Calabria, Campania, Molise, Puglia (Apulia), Sardegna, Sicilia. |
| --- | --- |

Figure S 1 Northern and Central-Southern Italian Italian Regions

| Gender Mortality  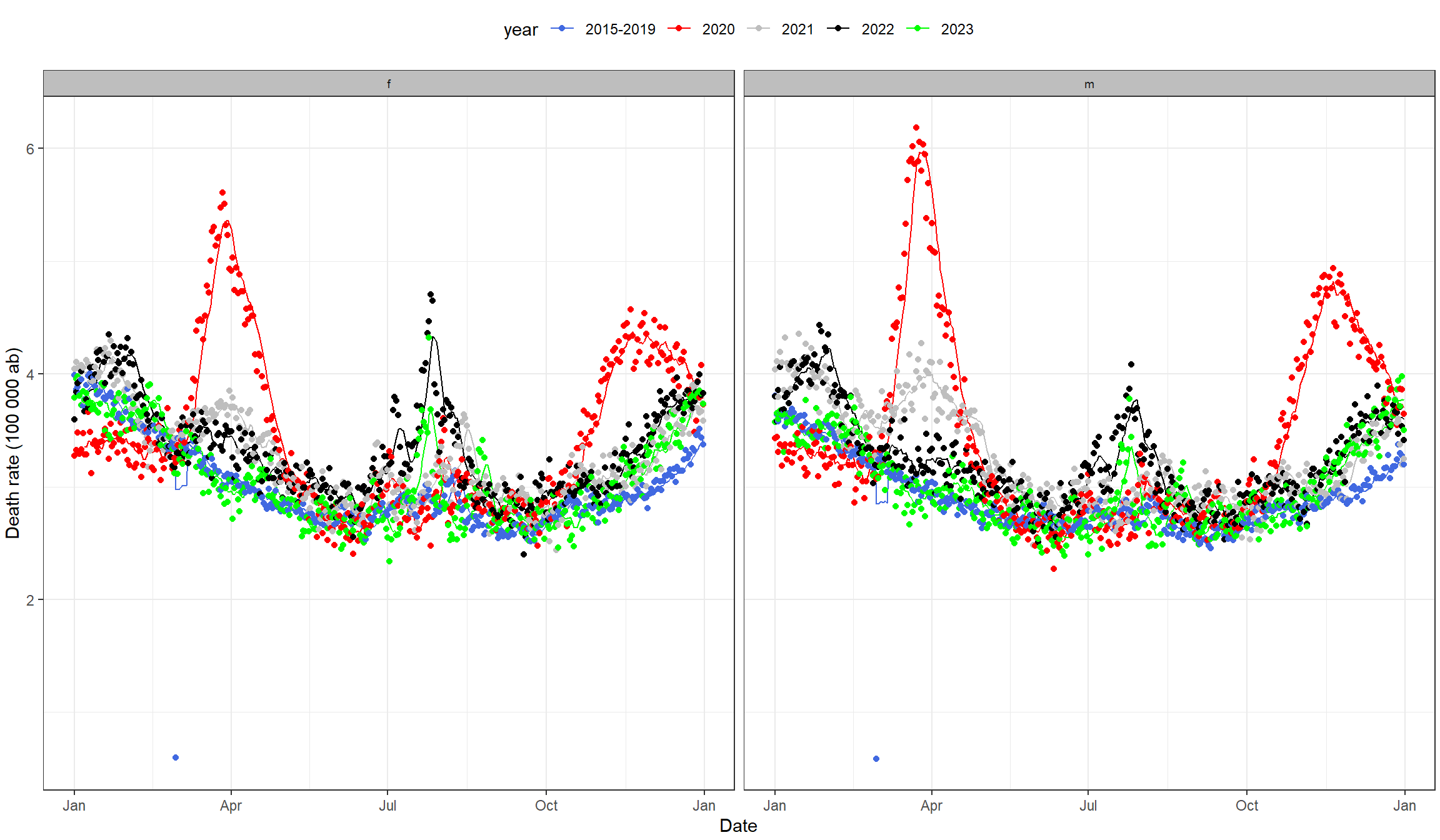  m = male; f = female |
| --- |

*Figure S2 Overall Mortality according to gender. Ab=Inhabitant.*

| Panel A Gender P-Score  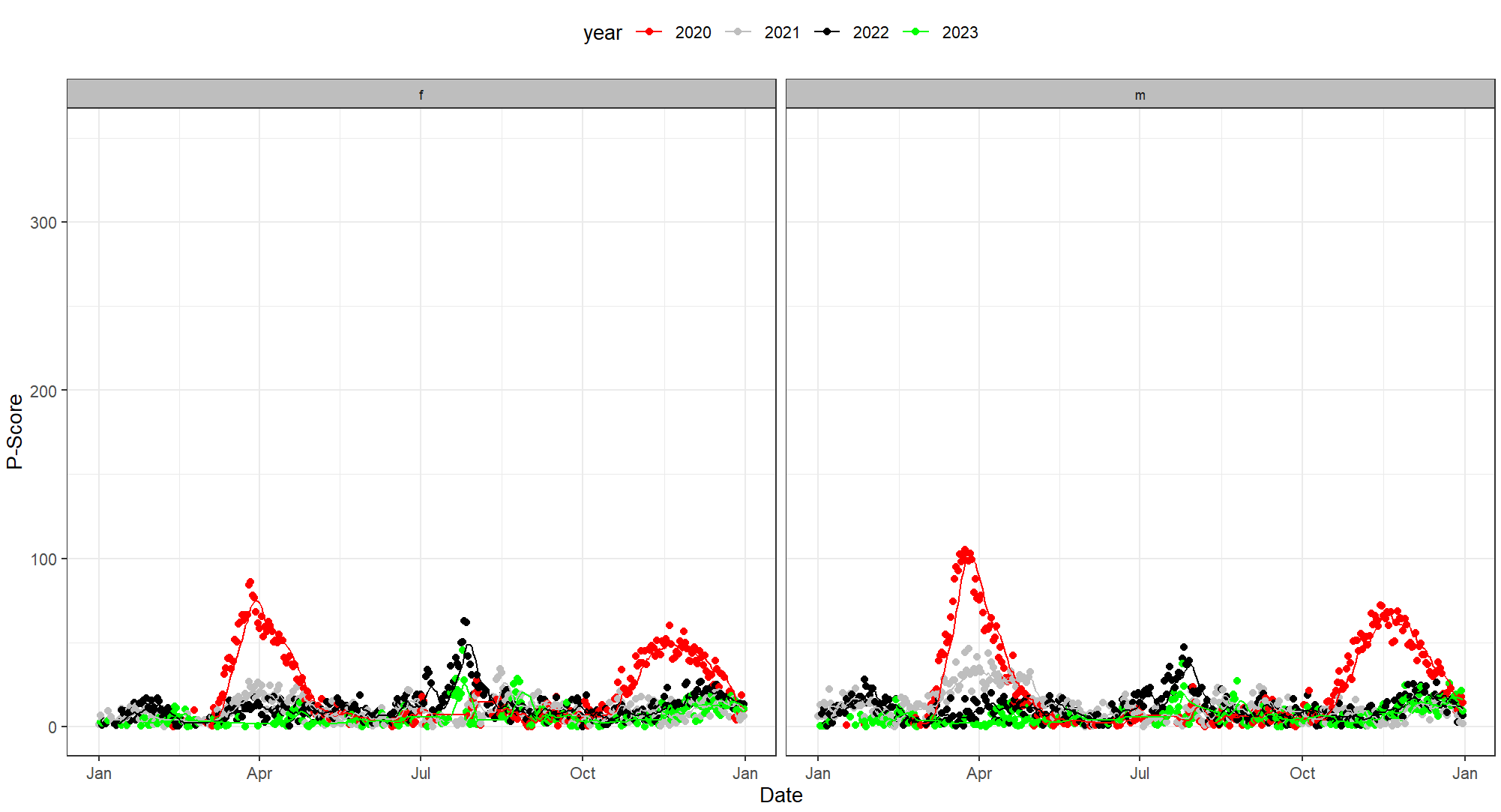  m = male; f = female |
| --- |

*Figure S3 P-Score according to gender.*

| Panel A Age Mortality  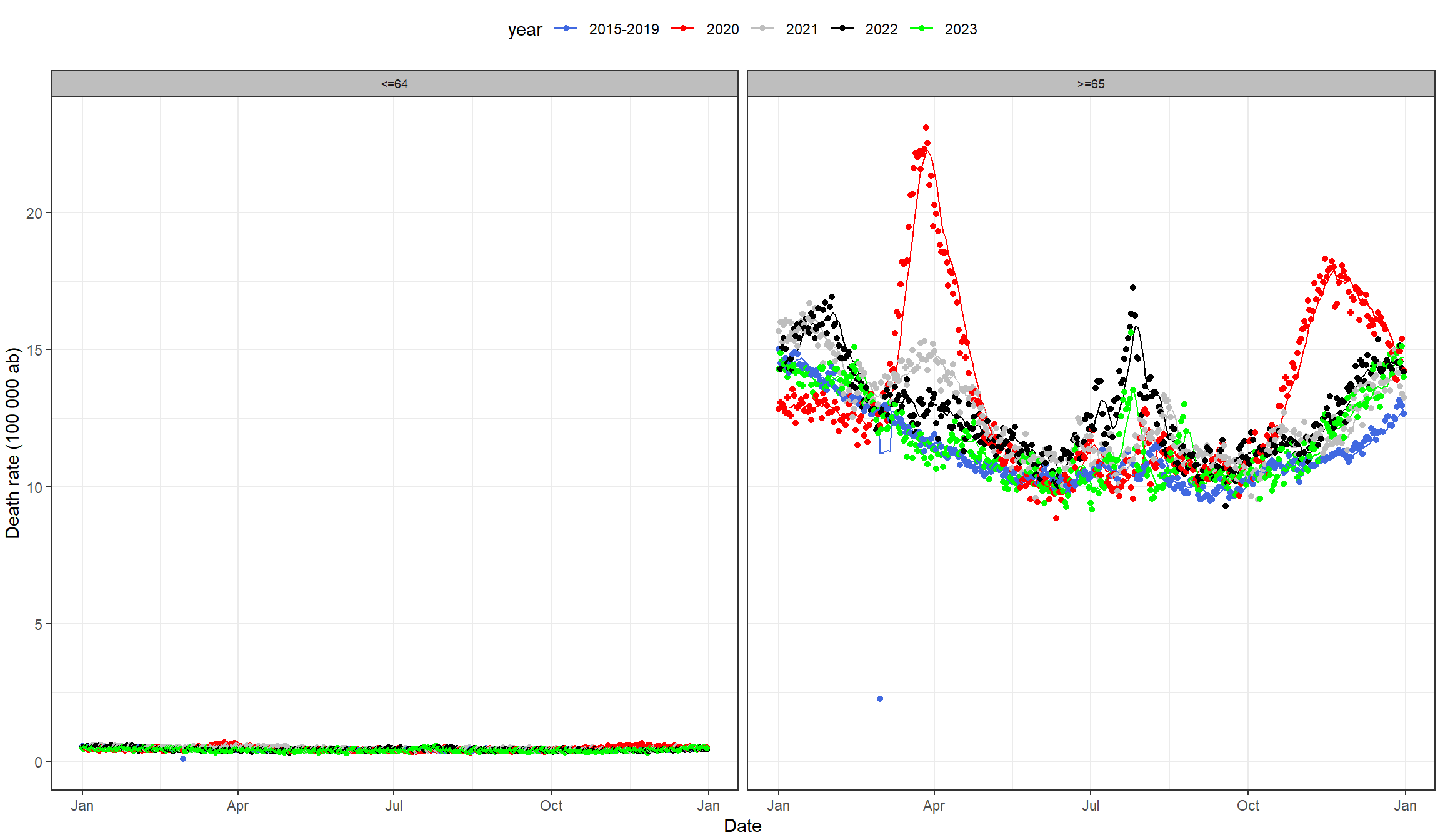 |
| --- |

*Figure S4 Overall Mortality according to age groups. Ab=inhabitants.*

| Age P-Score  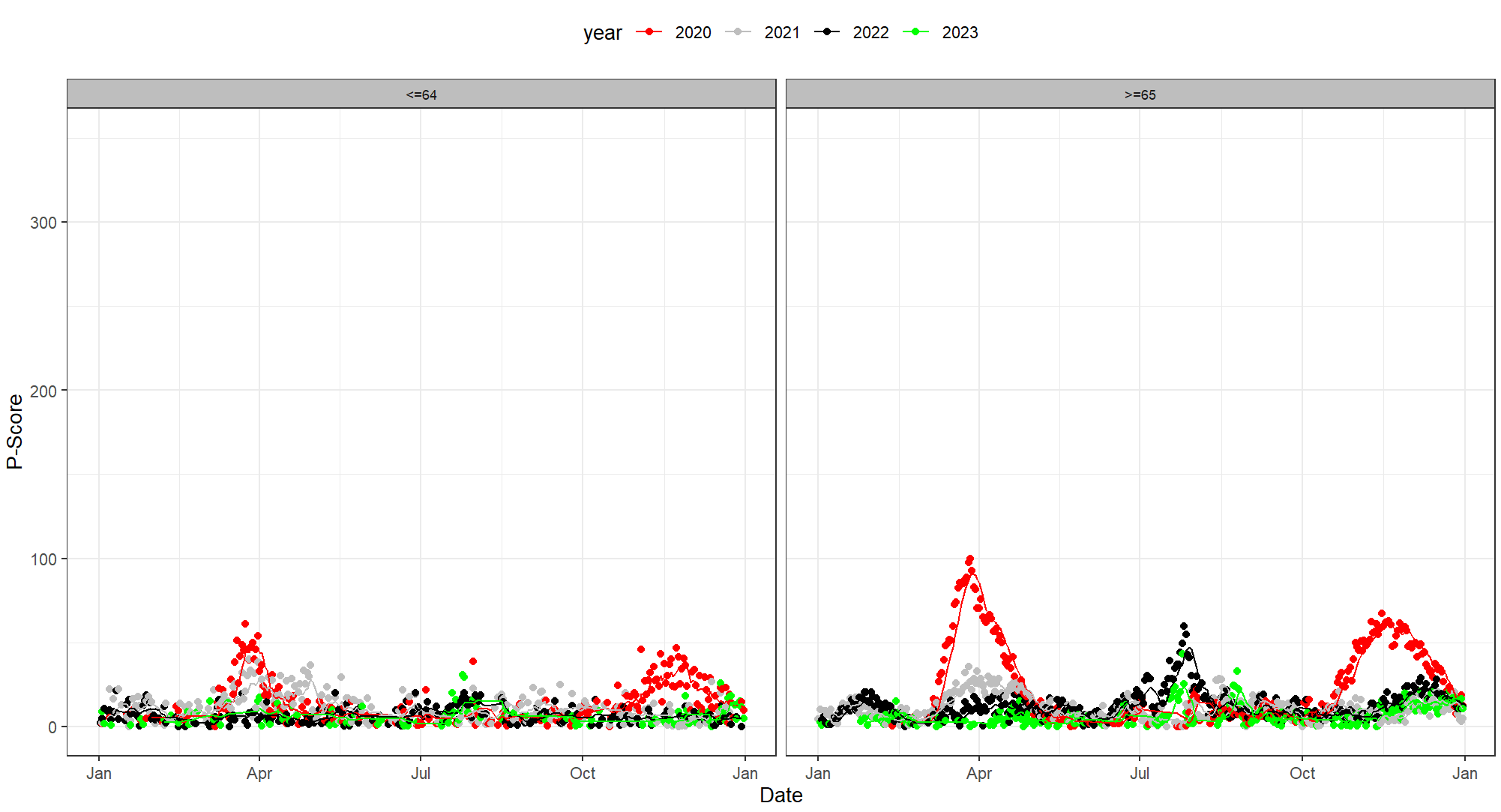 |
| --- |

*Figure S5 P-Score according to age groups.*
